# Improving the monitoring of chronic heart failure in Argentina: is the implantable pulmonary artery pressure with CardioMEMS Heart Failure System cost-effective?

**DOI:** 10.1186/s12962-021-00295-3

**Published:** 2021-07-09

**Authors:** Andrea Alcaraz, Carlos Rojas-Roque, Daniela Prina, Juan Martín González, Andrés Pichon-Riviere, Federico Augustovski, Alfredo Palacios

**Affiliations:** Health Technology Assessment and Health Economics Department, Institute for Clinical Effectiveness and Health Policy (IECS), Doctor Emilio Ravignani 2024, Buenos Aires, Argentina

**Keywords:** Heart failure, Pulmonary artery pressure monitoring, Cost-effectiveness, Argentina

## Abstract

**Background:**

The CardioMEMS® sensor is a wireless pulmonary artery pressure device used for monitoring symptomatic heart failure (HF). The use of CardioMEMS was associated with a reduction of hospitalizations of HF patients, but the acquisition cost could be high in low-and-middle income countries. Evidence of cost-effectiveness is needed to help decision-makers to allocate resources according to *“value for money”*. This study is aimed at estimating the cost-effectiveness of CardioMEMS used in HF patients from the third-party payer perspective -Social Security (SS) and Private Sector (PS)- in Argentina.

**Methods:**

A Markov model was developed to estimate the cost-effectiveness of CardioMEMS versus usual medical care over a lifetime horizon. The model was applied to a hypothetical population of patients with HF functional class III with at least one hospitalization in the previous 12 months. The main outcome was the incremental cost-effectiveness ratio (ICER). To populate the model we retrieved clinical, epidemiological and utility parameters from the literature, whilst direct medical costs were estimated through a micro-costing approach (exchange rate USD 1 = ARS 76.95). Uncertainties in all parameters were assessed by deterministic, probabilistic and scenario sensitivity analysis.

**Results:**

Compared with the usual medical care, CardioMEMS increased quality-adjusted life years (QALY) by 0.37 and increased costs per patient by ARS 1,081,703 for SS and ARS 919,051 for PS. The resultant ICER was ARS 2,937,756 per QALY and ARS 2,496,015 per QALY for SS and PS, respectively. ICER was most sensitive to the hazard ratio of HF hospital admission and the acquisition price of CardioMEMS. The probability that CardioMEMS is cost-effective at one (ARS 700,473), three (ARS 2,101,419,) and five (ARS 3,502,363) Gross Domestic Product per capita is 0.6, 17.9 and 64.1% for SS and 5.4, 33.3 and 73.2% for PS.

**Conclusions:**

CardioMEMS was more effective and more costly than usual care in class III HF patients. Since in Argentina there is no current explicit threshold, the final decision to determine its cost-effectiveness will depend on the willingness-to-pay for QALYs in each health subsector.

## Background

Heart failure (HF) is a public health concern due to its high prevalence and lethality, resulting in reduced life expectancy [1] and an impaired quality of life [2]. Although there is an increasing use of pharmacological [3–9] and intracardiac device [10–13] therapy options that showed improved morbidity and mortality, HF is still a major burden to patients, their caregivers and the national healthcare systems. Estimates from the USA suggest that the total cost of HF is US $31 billion and could increase to US $70 billion in 2030 [14].

Most of the costs are incurred from hospitalizations to treat clinical decompensations of patients with HF [15]. One of the most important causes of HF decompensation is the non-treatment adherence to medical indications, both regarding medication and non-pharmacologic therapies; early diagnosis in this scenario allows early treatment adjustment [16]. Implantable hemodynamic sensors find their support in the relationship between hemodynamic variables such as left ventricular or pulmonary artery pressure (PAP), with the subsequent appearance of symptoms, functional limitation and prognosis of the disease. Although the effectiveness of remote monitoring depend on the particular device and patients’ characteristics [17, 18], remote monitoring of PAP in patients with HF can enable medical professionals to access to additional pathophysiological information and improve decision-making to prevent hospitalizations and slow the progression of symptomatic HF [19–23].

The CardioMEMS sensor is a novel wireless PAP measurement. According to the results of the pivotal randomized controlled trial (CHAMPION) [21], the device can be considered for monitoring symptomatic patients to reduce HF hospital admissions and the economic burden [24]. CardioMEMS have been approved to be used in the USA by the Food and Drug Administration (FDA) [25, 26], and the Heart Failure Association of the European Society of Cardiology (ESC) included the device in the ESC guidelines for the diagnosis and treatment of acute and chronic HF [24]. Previous economic evaluations showed that CardioMEMS is cost-effective in the USA [27–29] and is likely to be cost-effective in the United Kingdom [30].

In Argentina, the use of CardioMEMS was approved in 2015 [31], but there is no evidence to assess the cost-effectiveness of devices for monitoring PAP. Evidence of the cost-effectiveness of HF management strategies is needed to help decision-makers to allocate scarce resources to address the mortality and morbidity burden, as well as to reduce the growing costs for patients and the healthcare system. Argentina is an interesting case of study due to the great fragmentation and segmentation of the health system [32]. Three large subsectors can be identified: the public, social security and private sector. The public sector covers 100% of the population, but approximately 36.5% (15.7 million of people) have exclusive public coverage [33, 34]. Social security is the most important sector within the health system, giving coverage to 60% (26 million) of the population. This sector is organized in three large groups: (i) 269 *Obras Sociales Nacionales* (OSNs) covering 14 million, generally composed of workers within the same labor activity and their core family members, (ii) 24 *Obras Sociales Provinciales* (OSPs) covering 7 million, generally composed of public employers, and (iii) the nationwide social health insurance fund for retired workers (PAMI, acronym in Spanish), covering 4.8 million elderly people and people with disabilities [33, 34]. Lastly, the private sector covers approximately 6 million, where 2 million are enrolled on an individual basis through direct and voluntary payment, while the remaining 4 million comes from the OSNs by contracting private supplementary plans [33, 34]. In addition, the high prevalence of HF (1–1.5%), especially in people older than 65 years in which prevalence is up to 8 times higher [16], adds relevance to the study.

This study is aimed at estimating cost-effectiveness measured through incremental costs per QALY of CardioMEMS compared to the usual medical care, from the third-party payer perspective in patients with HF functional class III in Argentina.

## Methods

### Decision model

We developed a state transition (i.e., Markov) model in Microsoft Excel® (Microsoft Corp. Redmond, WA) to determine the cost-effectiveness of the use of CardioMEMS HF system compared to the usual medical care over a lifetime horizon, from the third-party payer perspective, in particular, for Social Security (SS) and Private Sector (PS) in Argentina.

The Markov model was applied to a hypothetical population of 1000 patients with characteristics similar to the patients enrolled in the CHAMPION trial [21, 35, 36]. This was the latest available evidence of efficacy and safety of CardioMEMS while this study was ongoing. Thus, in the model we include patients with diagnosis of HF class functional (CF) III (according to the classification of the New York Heart Association (NYHA) [37]) regardless of the systolic ejection fraction, with at least one hospitalization for decompensation of HF during the previous 12 months. It was compared to 1000 hypothetical patients that received usual medical care consisting of optimized drug treatment and followed up according to the criteria of the attending physician.

The outcome of our model is the incremental cost-effectiveness ratio (ICER), which compares the difference in costs divided by the differences in Quality Adjusted Life Years (QALY) of CardioMEMS versus usual medical care. Nowadays, in Argentina, there is no explicit threshold to define an intervention as cost-effective, it is necessary to define a decision rule, defined as the willingness-to-pay for the health outcome that will be later used as a threshold. We adopted a willingness-to-pay threshold between 1 (ARS 700,473) and 3 (ARS 3,502,363) Gross Domestic Product (GDP) per capita based on decision rules estimated for low-and middle income countries [38–40], and previous economic evaluations published in Argentina [41–43] and based on the recommendation of the World Health Organization of 3 times the GDP [44]. Lastly, we explored a willingness-to-pay threshold of 5 GDP per capita since the technology is recommended for end-of-life care, for a disease associated with short life expectancy that would be extended thanks to the technology. The latter approach to consider a higher willingness-to-pay has been considered by the National Institute for Health and Care Excellence (NICE) in the United Kingdom upon establishing the criteria for the appraisal of end-of-life treatments [45]. We applied an annual discount rate of 5% for costs and QALY, as recommended by the economic evaluation guidelines for countries member of MERCOSUR [46], and we followed the CHEERS guidelines to report our findings [47].

### Model structure

**Fig. 1:**
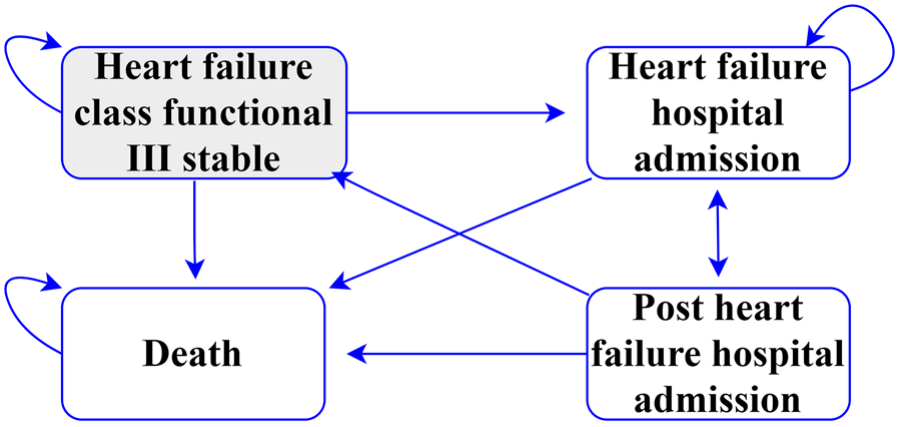
Analytic structure of the model. Gray square represents the initial state in the model

To define the model structure, we reviewed previously published specialized literature and economic models [27–30, 48]. Subsequently, we repeatedly resorted to the opinion of cardiology experts in order to validate the structure of the model in the local context. Our Markov model considered four health states: “Heart failure functional class III (stable)”, “HF hospital admission”, “Post HF hospital admission” and “Death” (Fig. 1). There are two cohorts in the model, those who receive the CardioMEMS device (treatment cohort) and those who receive the usual medical care (control cohort). For both cohorts, HF patients transitioned through different states in monthly cycles, accruing costs and QALYs. The length of the cycles in the model was defined as monthly because, during this time frame, the patients face high mortality and morbidity rates. The monthly cycles used are aligned with the previous economic evaluations performed [27–30, 48].

Before entering the model, the individuals in the treatment cohort accrue an initial incremental cost related to the implantation of the device and its complications (including acute mortality). We included the eight CardioMEMS complications reported in the Manufacturer and User Facility Device Experience (MAUDE) database, which collect mandatory and voluntary reports of device-related malfunctions, injuries or deaths received by the FDA [49]. In each cohort, patients enter to the model and may remain outpatient/stable, or they may require HF hospital admission or die. After a cycle, patients who entered the HF hospital admission state and are still alive enter the post HF hospital admission cycle and then return to an outpatient/stable cycle or require another hospital admission. Patients hospitalized and in the post HF hospital admission cycle have higher odds to die and accrue lower QALYs in comparison to patients who remain outpatient/stable.

Other assumptions were that the model did not consider non-HF hospital admission, because we assume they were equal in both cohorts. In addition, no adverse events associated with the pharmacological treatment received were considered, since the medication scheme was assumed to be the same in both cohorts.

### Epidemiological parameters

Every epidemiological parameter was retrieved by performing a literature search of the evidence regarding the patients and the intervention of the study. We select the evidence that suits the best and represents the local context. Epidemiological parameters are reported in Table 1.

**Table 1:** Summary of input parameters

Parameter	Base case	Variability range^a^	Probability distribution	Source
Clinical, efficacy and epidemiological parameters				
Baseline mean age of HF patients entering model	66	61 to 69	Normal	Weighted mean based on heart failure registries in Argentina
Baseline risk of HF hospital admission	1.13	0.68 to 1.55	Beta	Author estimations based on 21, 35, 36
HR of HF hospital admission, CardioMEMS cohort	0.48	0.38 to 0.67	Log normal	Meta-analysis based on 21, 35, 36
Baseline risk of mortality at 12 month of follow-up	0.16	0.14 to 0.18	Beta	35
HR of risk of mortality due to HF hospital admission	3.32	1.00 to 5.00	Log normal	51
Complications, treatment cohort (1–7 month)	0.03	0.02 to 0.04	Beta	49
Mortality due to device implantation procedure (1–7 month)	0.004	0.003 to 0.005	Beta	49
Utility parameters				
Baseline utility	0.711	0.027	Beta	21
Monthly change of utility for month 1 to 6, treatment cohort	0.001	0.001	Beta	21
Monthly change of utility for month 7 to 60, treatment cohort	0.003	0.003	Beta	21
Monthly change of utility for month 1 to 6, control cohort	−0.005	0.004	Beta	21
Monthly change of utility for month 7 to 60, control cohort	−0.003	0.003	Beta	21
Disutility of HF hospital admission	0.045	0.012	Beta	28
Costs parameters, in ARS				
Cost of CardioMEMS device	1,392,182	1,044,137 to 1,740,228	Normal	Abbott
Implant procedure	SS: 100,878 PS: 137,090	SS: 75,659 to 126,098 PS: 102,818 to 171,363	Normal	56
Weighted average cost of device complications^b^	SS: 144,482 PS: 193,445	SS: 108,362 to 180,603 PS: 145,084 to 227,954	Normal	56
Monthly device monitoring cost	SS: 973 PS: 1,502	SS: 730 to 1,217 PS: 1,126 to 1,877	Normal	56
Standard HF care cost	SS: 4,512 PS: 5,440	SS: 3,384 to 5,641 PS: 4,080 to 6,800	Normal	56
Hospital admission for HF cost	SS: 362,788 PS: 534,983	SS: 272,091 to 453,485 PS: 401,237 to 655,753	Normal	56–58

*PSA* Probabilistic sensibility analysis, *HF* heart failure, *HR* hazard ratio, *LMCCF* last mothly change carried forward, *SS* Social Security, *PS* Private Sector

^a^Varibility range for the clinical, efficacy and epidemiological parameters are reported as 95% confidence interval; variability range for the utility parameters are presented as standard deviation; variability range for the cost parameters are reported as minimum and maximum values

^b^The costs of the eight CardioMEMS complications were estimated as a weighted average according to its frequency

Exchange rate US $1 = ARS 76.95

The mortality for the patients in the treatment cohort at month 12 of follow-up was 16% (95% CI: 14–18%), based on the largest study published with the device: a multi-center, prospective, open-label, observational, single-arm trial, that assessed the efficacy and safety of CardioMEMS with 1200 patients [35]. The mortality for the control cohort was based on the life tables by age of the Argentine population and was calibrated through an intermediate correction factor that represents the excess of risk associated with HF in Argentina. The resulting correction factor, equal to 7.7, means that patients with HF CF III have an increased relative risk of death of 7.7 in comparison to those patients without HF. This correction factor for mortality was calculated by calibrating the baseline mortality rate of our model in the control cohort with the mortality rate observed in the control arm of the CHAMPION trial (23% at 18 months) [21].

The baseline mean age of patients entering the model was based on the weighted sample size mean age of patients with chronic HF reported in the Heart Failure Registries of Argentina [50]. To obtain the mean hazard ratio of heart failure hospital admission for patients in the treatment cohort, we performed a meta-analysis, including the CHAMPION trial [21] and two multi-center, prospective, open-label, observational single-arm trials that evaluated the efficacy and safety of CardioMEMS in patients with HF class III, in the USA and Europe [35, 36]. This meta-analysis was also used to obtain the mean of HF hospital admission events per patient-year. The decision of using all the internationally available evidence was made since in Argentina the Heart Failure Registry does not differentiate the rate of hospital admissions or the hospital admission events per person-year according to the classification of NYHA.

### Treatment effect of CardioMEMS

Due to the uncertainty of CardioMEMS regarding the direct effect on mortality, and since mortality in patients with HF increases during hospitalization and the subsequent month [51, 52], we indirectly modelled the effect on mortality through the decrease in the probability of HF hospital admission. We incorporated an increased risk of mortality [53] for HF hospital admission state and post HF hospital admission state (Table 1), a similar approach adopted by others [48]. CardioMEMS effect on HF hospital admission was modeled using the hazard ratio obtained in our meta-analysis, which included 3 studies [21, 35, 36]. For the base case analysis, these benefits were assumed to last five years, and disappeared after this period. This conservative decision was made due to the uncertainty regarding the long-term effects of the device, similar to a previous economic evaluation [30].

### Health-related quality of life

Data regarding baseline health-related quality of life (HRQoL) at 6 and 12 months was based on the EuroQol Quality of Life Five Dimensions instrument (EQ-5D) [54] and collected in the CHAMPION trial [21]. At baseline, the HRQoL is 0.711 for both cohorts, and at 6 and 12 months the HRQoL is 0.719 and 0.739 for the treatment cohort respectively, while for the control cohort the HRQoL at 6 and 12 months is 0.681 and 0.66, respectively. Based on this data, we calculated the monthly change of HRQoL and distributed it evenly over the intervals for months 1–6 and for months 7–60 for both cohorts. In the model, we assumed that the effect of CardioMEMS on HRQoL at month 12 is carried forward to month 60, and after that we assumed that the differences in utilities disappeared between both cohorts. This assumption is similar to the approach taken in previous economic evaluations [28]. The impact of HF hospital admission in utilities (or disutilities) was based on Schmier and colleagues [28]. We compared the Minnesota Quality of Life Questionnaire scores reported by CHAMPION trial with those reported in a local study [55], and the values were similar, suggesting EQ-5D scores could be applicable to Argentina.

### Direct medical costs

We applied a micro-costing approach to estimate the direct medical costs for a third-party payer perspective (SS and PS). Identification and quantification of healthcare resources were made by a local literature review, validated by consultation to local experts, whilst unit cost estimations were made using the Healthcare Cost Database of the Institute for Clinical Effectiveness and Health Policy (IECS) [56]. This database contains unit cost information based on the tariffs of medical resources and practices for SS and PS subsectors. Direct medical costs were updated to September 2020 and were expressed in Argentinian pesos (ARS) (exchange rate USD 1 = ARS 76.95).

The CardioMEMS device acquisition price was provided by the manufacturer and was ARS 1,398,182. The cost of CardioMEMS implantation was assumed to be equivalent to the cost of the right heart catheterization and angiography, a similar assumption made in another economic evaluation [27]. The costs of the eight CardioMEMS complications [49] were estimated as a weighted average according to its frequency. Death has no cost in the model. We consulted clinical experts to monetize the monthly cost of device monitoring.

Pharmacological treatment in the outpatient/stable cycle is based on the local clinical practice guidelines [16] and standard HF healthcare costs were estimated according to the local clinical practice guidelines for the treatment of HF [16]. The identification and quantification of the healthcare resources during the HF hospital admission in Argentina was based on two local studies [57, 58]. After this, we estimated the unit costs by using IECS Healthcare Cost Database [56].

### Sensitivity analysis

Uncertainty was assessed with one-way deterministic sensitivity analysis (Table 1). Due to the heterogeneity in the HF population, we assessed the baseline mortality risk, the baseline risk of hospital admission, the average age of HF population, and we also included the acquisition cost of the device. A probabilistic sensitivity analysis was performed using 1000 Monte Carlo simulations, in which, with each simulation, we sampled from the distributions of each input parameter. The range of values incorporated in these analyses is shown in Table 1.

### Scenario analysis

We assessed the uncertainty of CardioMEMS effect on patients through two analyses of alternative scenarios. In the first scenario, the treatment effect of CardioMEMS was extended to a lifetime horizon. In the second scenario, the effect of CardioMEMS lasts 18 months (equal to the follow-up time in the CHAMPION trial), and from this point, the effect progressively declines until the fifth year, when it finally disappears. Lastly, we also report the undiscounted base case results.

## Results

### Base case results

The parameters used in the model are reported in Table 1. In the base case analysis (lifetime horizon) with a discount rate of 5%, the CardioMEMS HF system increased QALYs in the treatment cohort in comparison to the control cohort by 0.37. CardioMEMS also increased costs compared to usual medical care by ARS 1,081,703 for SS and ARS 919,051 for PS (Table 2). The resultant ICER was ARS 2,937,756 per QALY and ARS 2,496,015 per QALY for SS and PS, respectively.

**Table 2:** Base case results

Strategy	Per patient cumulative costs (ARS)		Incremental costs (ARS)		Per patient cumulative QALY	Incremental QALY	Incremental cost-effectiveness ratio (ARS per QALY)	
	Social security	Private sector	Social security	Private sector			Social security	Private sector
Base case results with 5% discount rate								
Usual medical management	1,440,033	2,080,035			1.98			
CardioMEMS HF System	2,521,736	2,999,087	1,081,703	919,051	2.35	0.37	2,937,756	2,496,015
Base case results without discount rate								
Usual medical management	1,672,268	2,415,684			2.29			
CardioMEMS HF System	2,726,678	3,293,612	1,054,410	877,928	2.72	0.43	2,449,631	2,039,625

*HF* heart failure, *QALY* quality-adjusted life years

On the other hand, in the undiscounted base case analysis, CardioMEMS in comparison to usual medical care increased costs by ARS 1,054,410 and ARS 877,928 for SS and PS, respectively. QALY increased in the CardioMEMS cohort in comparison to the control cohort by 0.43. The resulting ICER was ARS 2,449,631 and ARS 2,039,625 per QALY for SS and PS, respectively.

### Sensitivity analysis

**Fig. 2:**
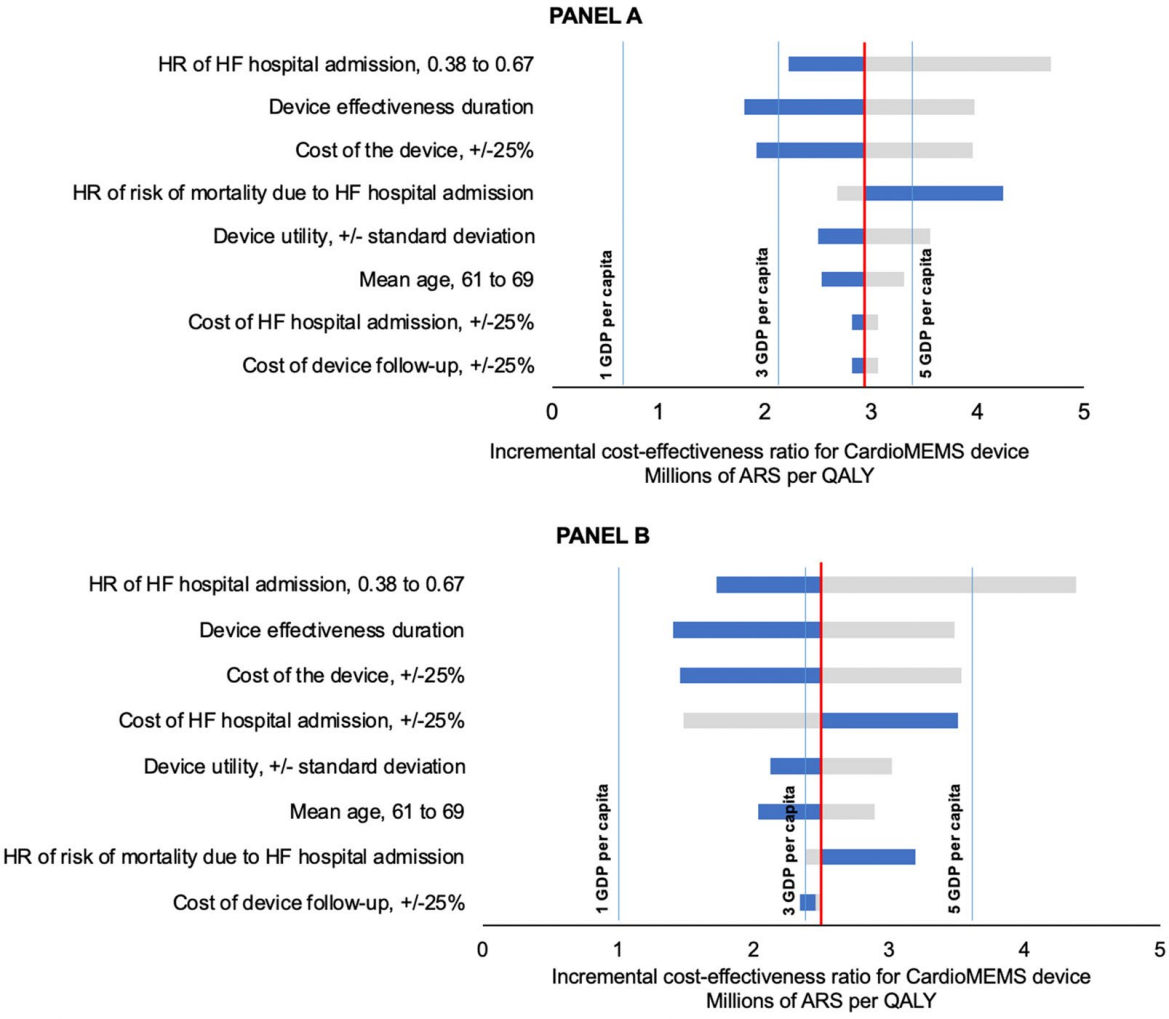
Tornado diagram: one-way sensibility analysis for the social security sector (**A**) and private sector (**B**). The bars indicate the range of ARS per QALY obtained with the CardioMEMS device compared to usual medical care in 1-way sensitivity analyses of the input parameters across the range of values. The solid red line represents the base case cost-effectiveness result of ARS 2,937,756 per QALY gained for the social security sector and ARS 2,496,015 for the private sector. Blue lines at one, three and five GDP per capita ARS 700,473, ARS 2,101,419 and ARS 3,502,363, respectively. *HF*, heart failure; *HR*, hazard ratio

Figure 2 shows the one-way sensitivity analyses performed for both the SS and PS perspective. The ICER was most sensitive to the HR of HF hospital admission obtained in our meta-analysis, the acquisition cost of CardioMEMS and the mortality due to HF hospital admission, among other parameters. Reducing the HR of HF hospital admission to 0.38 [the base HR reported in Angermann et al. [36]] resulted in an ICER of ARS 2,225,077 and ARS 1,728,223 per QALY for SS and PS, respectively. On the other hand, increasing the HR to 0.67 (the HR reported in the CHAMPION trial [21]) increases the ICER to ARS 4,685,811 and ARS 4,382,352 per QALY for SS and PS, respectively. The ICER was also sensitive to the acquisition cost of the device. When we reduced the acquisition cost of CardioMEMS by 25%, ICER resulted in ARS 1,924,020 per QALY for SS and ARS 1,457,693 for PS. A discount of 10% in the acquisition cost of CardioMEMS yields an ICER below 3 GDP per capita.

Another parameter that influenced the ICER result was HR of risk of mortality due to HF hospital admission (varied between 1 and 5). The resulting ICER varied from ARS 2,683,044 to ARS 4,235,255 per QALY for SS and varied from ARS 2,379,068 to ARS 3,193,044 per QALY for PS. Lastly, when we varied the utility of CardioMEMS by its standard deviation, the ICER varied from ARS 2,503,120 to ARS 3,555,040 per QALY for SS and varied from ARS 2,126,734 to ARS 3,020,485 for PS. ICER was fairly less sensitive to the variabilities in the rest of the parameters.

We also assessed the uncertainty of the CardioMEMS effect on patients by performing two scenario analyses. Under the first scenario, with a lifelong lasting CardioMEMS effect, the resulting ICER was ARS 1,804,312 per QALY for SS and was ARS 1,407,515 per QALY for PS. Under the second scenario, with a CardioMEMS effect of 18 months and henceforth progressively declining to completely disappear on year five, the resulting ICER was ARS 3,966,795 per QALY for SS and was ARS 3,484,989 per QALY for PS.

### Probabilistic analysis

**Fig. 3:**
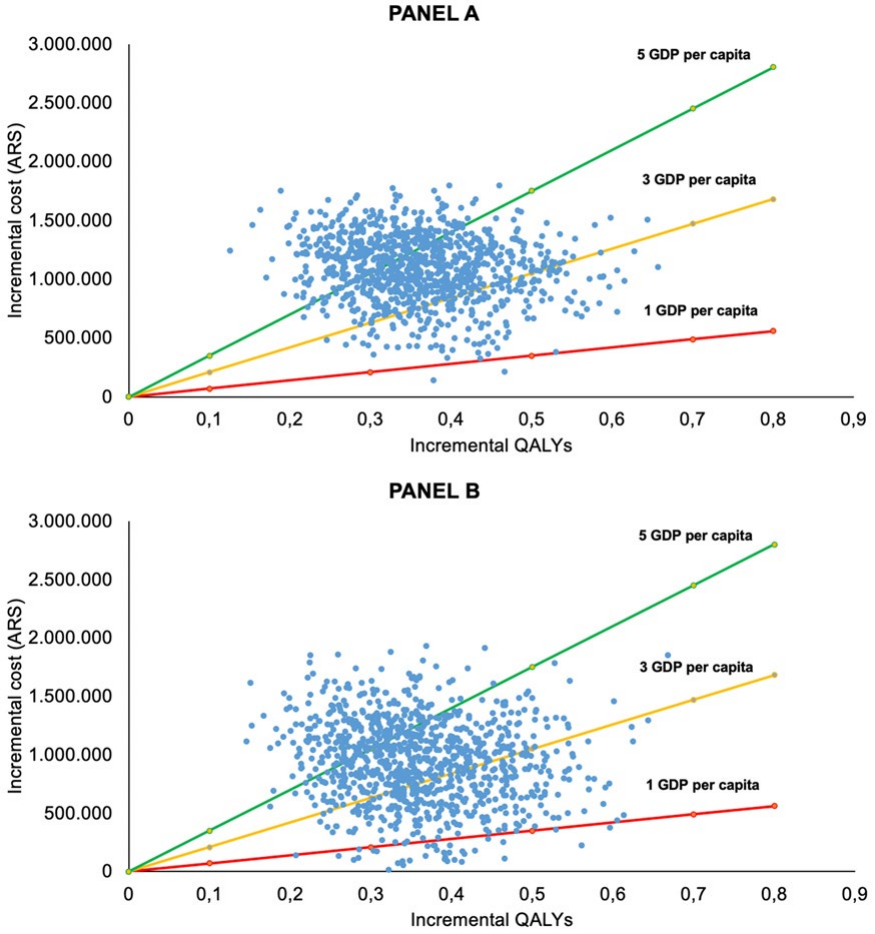
Cost-effectiveness scatter plot for social security sector (**A**) and private sector (**B**), for different willingness-to-pay thresholds. One GDP per capita (ARS 700,473), three GDP per capita (ARS 2,101,419), and five GDP per capita (ARS 3,502,363). Each point in the scatter plot represents one simulation in the model with different input values sampled from the input distribution

Figure 3 shows the results of the probabilistic sensitivity analysis. For SS, all the points in the scatter plot fall in the right upper quadrant, confirming the high certainty that CardioMEMS is both more effective and more expensive than the usual medical care. These findings are quite similar for the PS sector. For both sectors, at one GDP per capita as a cost-effectiveness threshold almost the totality of the points are above the threshold. On the other hand, at five GDP per capita as a cost-effectiveness threshold, the majority of the points in both sectors fall below the curve.

**Fig. 4:**
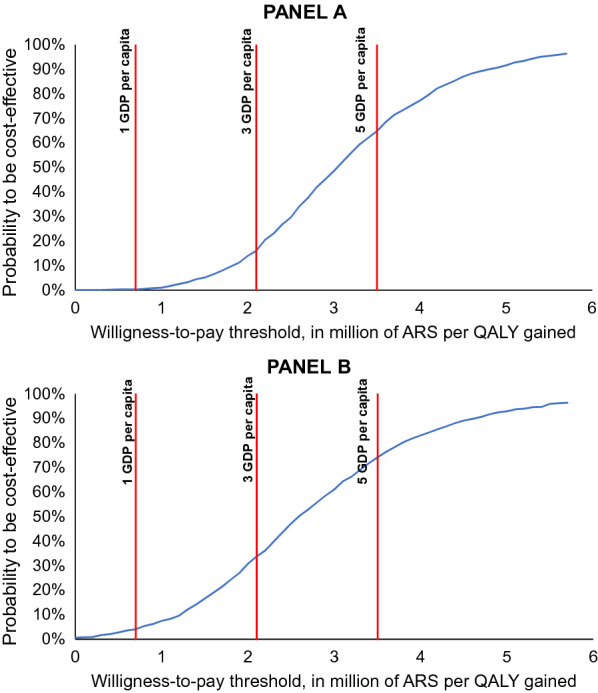
Cost-effectiveness acceptability curve of CardioMEMS device versus usual medical care for the social security sector in Argentina. Willingness-to-pay thresholds at one (ARS 700,473), three (ARS 2,101,419) and five (ARS 3,502,363) GDPs per capita

Due to the potential heterogeneity in willingness-to-pay among different third-party payers from the same sectors in Argentina, we assessed several thresholds of willingness to pay. For the SS sector, the probability that CardioMEMS be cost-effective at one (ARS 700,473), three (ARS 2,101,419,) and five (ARS 3,502,363) GDP per capita is 0.6, 17.9 and 64.1%. For the PS sector, the probabilities are 5.4, 33.3 and 73.2%, respectively (Fig. 4).

## Discussion

In contexts where resources for healthcare are scarce, health technologies assessments and economic evaluations are useful tools to evaluate the efficacy, safety, efficiency, and affordability of new healthcare technologies. From the efficacy and safety evidence of CardioMEMS HF system, this study assessed the cost-effectiveness of CardioMEMS use in the Argentinian context. The analysis showed that base case ICER was between three to five GDP per capita per QALY gained. Although Argentina does not have an explicit cost-effectiveness threshold, this result could be above the efficiency threshold suggested by the literature [46, 47]. However, considering that the benefited population comprehends patients with advanced-stage HF and poor prognosis, the decision rule to determine cost-effectiveness of the device will depend on the willingness to pay for QALYs gained from each specific third-party payer in Argentina.

It is important to mention that in Argentina, as in other Latin American countries, health technology coverage decisions are not necessarily guided by cost-effectiveness criteria. For example, Pichon-Riviere and colleagues estimated the ICER for trastuzumab, a breast cancer drug available in several Latin American countries, and showed that for Argentina trastuzumab only could be cost-effective at a threshold of 8.47 GDP per capita [59].

Evidence of cost-effectiveness analysis suggests that CardioMEMS could be cost-effective in the USA and UK settings, even though ICER varies widely due to the different time horizons adopted, the assumptions made in the effects of CardioMEMS on mortality, among other methodological aspects [27–30, 48]. The studies that incorporated a lifelong time horizon (as ours) showed a higher ICER than studies with a shorter analytic horizon, mainly due to the fact that the benefit of CardioMems declined through time. In our estimations, survival years, QALYs and HF hospital admissions averted were consistent with the estimations made by the studies that use lifetime horizons [27, 48].

In our model, among all parameters, ICER estimates were most sensitive to the HR of HF hospital admission. In the base case, to synthesize all the available evidence regarding the effectiveness of CardioMEMS on the hospital admission, we performed a meta-analysis using the pivotal trial [21] and two real-world evidence studies [35, 36]. In the one-way sensitive analysis we assessed the variability of this parameter by using the HR reported in Angermann and colleagues [36] and the HR reported in the CHAMPION trial [21], as they represent the extreme values in the available literature. In addition, we assessed all the treatment effects of CardioMEMS by performing two analyses of scenarios. In the best scenario where the treatment effect of CardioMEMS was lifelong, ICER fell below the 3 GDP per capita willingness-to-pay, but in the worst scenario where the treatment effect declined from month 18 and equal for both cohorts in month 60, ICER exceeded the 5 GDP per capita willingness-to-pay. We consider that our model incorporates a credible scenario for the base case, given that it is not possible to know whether the benefit of avoiding hospitalizations would continue as the patients get worse given that it is a progressive disease [60–62].

The results of the CHAMPION trial regarding the treatment effect of CardioMEMS on mortality were inconclusive [21], and studies of non-invasive remote monitoring systems have been neutral regarding the potential reduction in mortality [63, 64]. Due to this uncertainty, our approach in the modelling was to capture this treatment effect indirectly from the increased risk of mortality during HF hospital admission and the following month. The expected benefit on QALYS could be higher (and the ICER lower) if the reduction in mortality risk is confirmed. This approach is more conservative than the one used in other economic evaluations [28–30], but is similar to the approach adopted in an economic evaluation performed in the USA [48]. The lack of proven effectiveness in mortality still has to be weighed against the benefits in quality of life and cost from the decrease in hospitalizations by decision-makers.

Our findings could have implications on CardioMEMS pricing policies. As the largest costs of the model are driven by the acquisition cost of CardioMEMS, we varied this parameter by ± 25%. In the lowest price, ICER improves and falls below the 3 GDP per capita willingness-to-pay threshold on social security and a discount of 10% turns the ICER to 3 GDP in the private sector. This information together with budget impact analysis, is helpful to design pricing policies in the different health sectors in Argentina.

This study has some limitations to note. First, all the effectiveness and safety of CardioMEMS comes from a CHAMPION trial [21] and two real world studies [35, 36] that assessed effectiveness and safety at one year post implantation of CardioMEMS, thus the long-term efficacy is still unclear. To reduce the uncertainty, we performed a meta-analysis to summarize the best evidence available at the moment. In the near future, this meta-analysis should be updated as more post-surveillance evaluations become available. Second, the third-party payer perspective of this study prevented us from evaluating all the possible benefits and costs at a societal level. For example, reductions in the rate of HF hospital admissions could reduce indirect cost for patients (labor productivity loss costs, out-of-pocket expenditures) and favors medical attention to other HF and non-HF patients by reducing waiting time queue. Third, our model does not include some aspects of indirect saving that can improve the ICER, but there is a lack of information. For example, the time spent monitoring CardioMEMS, in spite of not influencing ICER results, is an important aspect to consider. CardioMEMS HF system seems plausible to require less time to monitor the device in comparison to other less advanced technologies such as telephone calls, anamnesis on body weight, diuretic rhythm and symptoms of dyspnea and fatigue. Fourth, we used a linear trajectory of utilities or disutilities; however, the relationship between patient-reported utilities and HF hospital admission may not be linear, since patients with multiple HF hospital admissions during an interval of time can report higher disutilities. Unfortunately, there is no data available to describe the utilities of HF patients regarding hospital admissions, but in our sensitivity analysis we widely assessed this parameter and did not show greater impact on ICER. Given the substantial societal and economic toll of HF, it is worthy to consider the previous aspects as soon as they become available. Incorporating these types of devices into the coverage package should take into account other strategies to improve patient adherence to treatment at the same time [65].

Despite these limitations, this study gives a clear snapshot about the cost-effectiveness of CardioMEMS in Argentina, underpin the finding with a model adapted to the local setting and using a nationally representative costs database to perform a micro-costing approach to estimate costs of health events. Furthermore, the sensibility analysis was useful to examine the multiple variability of ICER, thereby findings herein presented can promote the use of cost-effectiveness evidence in HF management strategy adoption at a national level. Future studies that refine estimates of the long-term effects of the device on mortality could reduce uncertainty and improve conclusions regarding its clinical and economic impact, contributing to informed healthcare decision-making.

## Conclusion

CardioMEMS was more effective and more costly than usual care in class III HF patients in Argentina, being the ICER between three and five GDP per capita per QALY gained. The decision rule to determine the cost-effectiveness of the device will depend on the specific willingness to pay for QALY gained from each healthcare subsector.

## Data Availability

The datasets analyzed in this study are available from the corresponding author on reasonable request.
